# Spatial Regulation of Epidermal Growth Factor Receptor Signaling by Endocytosis

**DOI:** 10.3390/ijms14010072

**Published:** 2012-12-20

**Authors:** Brian P. Ceresa

**Affiliations:** Department of Pharmacology and Toxicology, University of Louisville, Louisville, KY 40202, USA; E-Mail: brian.ceresa@louisville.edu; Tel.: +1-502-852-2564; Fax: +1-502-852-7868

**Keywords:** epidermal growth factor receptor, endocytosis, signal transduction

## Abstract

Signaling by cell surface receptors appears to be relatively straight-forward: ligand binds to the extracellular domain of the receptor and biochemical changes are communicated into the cell. However, this process is more complex than it first seems due to the various mechanisms that regulate signaling. In order to effectively target these receptors for pharmacological purposes, a more complete understanding of how their signaling is regulated is needed. Here, how the endocytic pathway regulates receptor signaling is discussed, using the epidermal growth factor receptor (EGFR) as a model. In particular, the spatial regulation of signaling is examined. Areas of discussion include: how endocytic trafficking affects biology/pathology, varying approaches for studying the relationship between receptor endocytosis and signaling, and developments in how the endocytic pathway controls EGFR:effector communication and EGFR-mediated cell biology.

## 1. Introduction

The epidermal growth factor receptor (EGFR) is arguably the most well studied and characterized receptor tyrosine kinase. Activation of the EGFR is critical in embryonic development [1], tissue homeostasis [2] and wound healing [3]. These physiological events are evident by the embryonic lethality of EGFR knock-out animals [4] as well as tissue defects in EGFR ligand knock-out animals [5]. In addition, many cancers are characterized by overexpression and/or mutations that hyperactivate the EGFR [6]. Therefore, a complete understanding of how the EGFR functions has important implications in developmental biology, tissue repair, and cancer biology.

Like all cell surface receptors, the EGFR communicates changes on the outside of the cell to within by biochemical signals via effector proteins. Cell physiology, such as proliferation, differentiation, and migration, is governed by the coordinated activity of these effectors. Binding extracellular ligands is clearly the mechanism for initiating these signaling pathways. However, the details regarding how subsequent receptor activity is controlled is not as straightforward.

Numerous mechanisms impact receptor signaling, including the concentration of ligands available to bind receptors, the density of receptors, the duration for which the receptor stays activated, and the proximity of the receptor to downstream effector molecules. Understanding the mechanisms that regulate EGFR signaling will aid in developing therapeutic compounds for the treatment of various maladies, such as cancer and wound healing. In this review, we will examine one cellular process that has emerged as a key regulator of EGFR signaling: the endocytic pathway. Although the discussion will focus primarily on EGFR membrane trafficking and signaling, these findings have parallels with other cell surface receptors, particularly other receptor tyrosine kinases.

## 2. The Endocytic Pathway

Internalization of cell surface receptors can be divided into clathrin-independent and clathrin-mediated endocytosis, depending on the route of entry into the cell. Clathrin-independent endocytosis includes internalization via caveolae or various small molecular weight GTP binding proteins (*i.e*., Arf6, RhoA, CDC42) [7] and is associated with higher EGF concentrations (20 ng/mL) [8]. Clathrin-mediated endocytosis of the EGFR occurs with lower concentrations (1.5 ng/mL) of EGF [8]. One plausible explanation for the differing, concentration-dependent routes of ligand:receptor complex entry is that the primary pathway becomes saturated and alternative mechanisms are employed. Saturation of the endocytic pathway has been reported at more distal stages of the pathway when higher levels of the receptor are expressed [9,10].

### 2.1. Components of the Pathway

**Figure 1:**
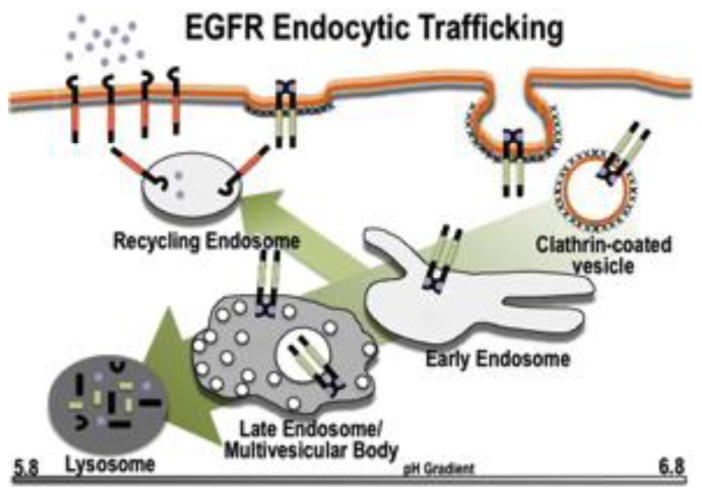
Schematic of the Endocytic pathway. Highlighted is the major route of epidermal growth factor receptor (EGFR) endocytic trafficking—movement of the ligand:receptor complex to the lysosome for degradation. Also shown is recycling of the EGFR back to the plasma membrane. Alternative routes of trafficking as discussed in the text.

The basic events associated with ligand-stimulated, clathrin-mediated endocytosis have been fairly well delineated for some time [11], although new molecular details continue to emerge (Figure 1). Briefly, the liganded EGFR monomers dimerize and translocate along the plasma membrane until it associates with a membrane domain that is enriched with clathrin on the intracellular face. This domain invaginates to form a clathrin-coated pit, which pinches off forming a clathrin-coated vesicle. The clathrin is shed from this vesicle to produce an intermediate vesicle that fuses with and delivers the EGF:EGFR complex to the early endosome. In the early endosome, the ligand:receptor complex is readied for its ultimate cellular fate—either (1) the early endosome matures into a late endosome and delivers the cargo to the lysosome [12]; (2) a recycling endosome pinches off the early endosome and the ligand and receptor recycle back to the plasma membrane [13]; or (3) an endosome forms to deliver the receptor to some other intracellular organelle (*i.e*., mitochondria [14], trans-Golgi network [15], and endoplasmic reticulum [16]). It should also be noted that endocytic trafficking is distinct from the movement of the EGFR and EGFR fragments to the nucleus [17,18]. It is not entirely clear which cellular components determine the trafficking itinerary, although there is strong evidence that the stimulating ligand, receptor density, and cell type each have a contributing role.

Each endocytic route has a very different consequence on EGFR signaling. Trafficking to lysosomes will result in attenuated signaling due to receptor degradation. Receptors that recycle back to the plasma membrane have the opportunity to be re-stimulated by extracellular ligands that are present. When the ligand:receptor complex traffics to other subcellular organelles, receptor:effector interactions may occur due to enhanced localized effector concentrations that impact a defined cell biology. When a cell’s normal trafficking itinerary is perturbed, the duration, magnitude, and specificity of receptor signaling will be altered and can affect cell biology.

Based on many of the early studies, EGFR endocytosis was thought to be strictly a mechanism to control how long a liganded receptor stayed active. However, for almost 20 years, it has been appreciated that the endocytic pathway provides spatial regulation of signaling. Work by Vieira *et al.* that examines endocytosis-deficient EGFRs indicated that for some effectors, maximal activity requires that the receptor be internalized [19]. Thus, the endocytic pathway can be a positive regulator of signaling as well.

### 2.2. Regulators of EGFR Endocytosis

The movement of cargo through the endocytic pathway is coordinated at each stage by a number of proteins. This process is mediated by adaptor proteins that directly interact with the EGFR and the endocytic machinery (*i.e*., Grb2 and Eps8-[20]) and regulatory proteins that control movement between endocytic compartments. Many of the fission and fusion reactions that permit the transfer of cargo are mediated by guanine-binding proteins (G-proteins), that alternate between active, GTP-bound and inactive, GDP-bound states. Most of these proteins are members of the RAB family of small molecular weight G-proteins that are 20 to 25 kDa in size. Examples include RAB5 (plasma membrane to early endosome), RAB7 (early to late endosome maturation; late endosome to lysosome), RAB9 (late endosome to trans-Golgi Network), RAB11, RAB4, RAB25 (recycling to the plasma membrane) (Reviewed in [21,22]). A notable exception to the small molecular G-proteins is the large G-protein, dynamin, which promotes the fission of clathrin-coated pits from the membrane [23].

In addition to the requisite signaling events that ligand binding triggers and are associated with endocytosis, ubiquitylation is a key post-translational modification that directs the EGFR to lysosomal degradation [24,25]. The exact mechanism of ubiquitylation in EGFR has been controversial. There is general acceptance that EGFR ubiquitylation is mediated by the E3 ubiquitin ligase c-Cbl with associated with phosphotyrosine 1045 of the liganded EGFR [26,27]. Conflict arises over whether ubiquitylation regulates EGFR endocytosis [28,29] or targeting to intraluminal vesicles [30,31]. Such details regarding location are very important in membrane trafficking as they often provide important clues regarding function.

On one hand, there are reports that EGFR-Ub chimeric proteins have an accelerated receptor endocytosis [32]. On the other, mutant EGFRs with all lysines mutated such that it can not be ubiquitylated have no change in the rate of endocytosis [33]. These data, along with others [8,34], point to that ubiquitylation being sufficient, but not necessary for endocytosis. This review acknowledges, but does not attempt to resolve, this controversy.

Identification of these modifications and regulatory proteins has been critical in understanding how EGFR signaling is spatially regulated. These regulatory proteins and protein modifications are often antagonized, through dominate negative mutants or RNAi, as a strategy for disrupting trafficking at a specific endocytic stage.

## 3. Clinical Relevance of EGFR Endocytic Trafficking

Endocytic regulation of EGFR signaling is not entirely academic; there are implications in furthering our understanding of diseases. Changes that slow the progression of the EGFR through the endocytic pathway, enhance signaling, and can lead to increased cell proliferation, differentiation, migration, as well as other hallmarks of cancer [35]. For instance, a subset of non-small cell lung carcinomas are characterized by mutant EGFRs that preferentially recycle rather than degrade as the non-mutant EGFRs do. This leads to enhanced signaling via the proto-oncogene tyrosine kinase Src [36]. Conversely, accelerated trafficking may decrease the basal level of EGFR signaling and cause defects in tissue homeostasis (*i.e*., epithelial erosions or inflammations).

A better understanding of receptor trafficking may help in the treatment of both cancer and restoration of tissue homeostasis (wound healing). For instance, experimental administration of EGF to corneal epithelial cells clearly promotes healing with *in vitro* and *ex vivo* models, but yields inconsistent results in patients. This has impeded the use of EGF therapeutically. It has been suggested that prolonged agonist stimulation causes desensitization and attenuated EGFR signaling via lysosomal degradation of the agonist:receptor complex [25]. *In vitro* data indicate that diverting the activated EGFR from lysosomal degradation leads to enhanced corneal epithelial cell migration [37]. These data indicate that pharmacological inhibitors of the endocytic pathway are a potential mechanism for enhancing EGFR signaling in the cornea.

In both cancer and wound healing, targeting EGFR endocytic trafficking is a viable pharmacological strategy. However, from these two disparate examples, it is clear there are details that need to be worked out. Accelerating EGFR trafficking is predicted to decrease the growth factor-dependent survival of a cancer cell, but could disrupt epithelial cells homeostasis in other tissues. Conversely, decreasing trafficking would promote the signaling necessary for corneal wound healing, but could lead to hyperactivation of the EGFR and proliferation of other cells in the body. These examples illustrate how we need a better understanding of the molecular interplay between EGFR trafficking and signaling to minimize any unwanted side-effects.

## 4. Models for Studying EGFRs Deficient in Endocytic Trafficking

The spatial and temporal regulation of signaling are tightly intertwined and separating the two processes has been systematic and methodic. The differences in EGFR signaling under conditions of enhanced recycling *versus* increased degradation are obvious. However, the consequences of altering the subcellular localization of the ligand:receptor have been more complex. The molecular events that occur from subcellular locations can be difficult to dissect due to the dynamic nature of intracellular compartments and the intrinsic compensatory mechanisms of the cell. Nevertheless, there has been a continuum in understanding how trafficking is regulated, to identifying differences in receptor:effector communication at various endocytic stages, and more recent identification of cell biology that requires a specific subcellular localization of the activated EGFR.

To date, these studies have been primarily restricted to *in vitro* model systems, but the field is poised to move the molecular findings to more complex systems. As in most scientific research, movement toward more complex model systems, results in fewer molecular details. However, recent reports using model organisms demonstrate this can be done [38,39].

Even with *in vitro* model systems, studying the relationship between EGFR signaling and endocytosis is inherently difficult for a number of reasons. First, the two processes are dependent on one another—changes in signaling affect trafficking and altering trafficking affects signaling. Therefore, primary *versus* secondary effects can be difficult to assess. Second, endocytosis provides both temporal and spatial regulation of signaling. When endocytosis is disrupted and signaling is altered, without proper experimental design, it can be difficult to discern if the effects are due to the prolonged activation of the EGFR or due to enhanced receptor:effector interactions at a specific subcellular location. Third, it is unlikely the endocytic pathway is a “switch” in signaling, but rather a “rheostat”. Protein:protein interactions that are governed by the Law of Mass Action and any two proteins can interact, given sufficiently high local concentrations. Therefore, in order to draw biologically relevant conclusions, model systems need to be carefully chosen to reflect appropriate receptor and effector densities, ligand concentrations, and kinetic analysis. Using systems with inappropriately high levels of activated receptor, effector densities, or for extended periods of time, may overlook important spatial regulators of signaling.

There have been three basic strategies for studying the relationship between endocytic trafficking and receptor signaling: (1) receptor mutants, (2) inhibition of the endocytic pathway, and (3) ligands that alter trafficking. Before discussing recent findings, the strengths and weaknesses of each approach will be discussed.

### 4.1. Receptor Mutants

One of the most productive strategies for understanding the relationship between EGFR endocytic trafficking and signaling has been to mutate the receptor such that the trafficking, but not signaling, is compromised. The approach has been useful for identifying domains necessary for specific aspects of endocytic trafficking, identifying subcellular compartments, as well as the relationship between endocytosis and signaling [9,30,40,41].

One of the first examples of this strategy was by Wells *et al.* to make a truncation mutant of the EGFR that was endocytosis deficient, but still phosphorylated and signaling competent [41]. This group found that cells expressing these mutant receptors are more sensitive to EGF in cell transformation assays. More recently, lysine null mutants of the EGFR that cannot be ubiquitylated were shown to be trafficked away from the intraluminal vesicle of the late endosome and spared from lysosomal degradation. This mutation resulted in an increase in the magnitude and duration of EGFR phosphorylation and signaling to Extracellular Regulated Kinase 1/2 (ERK 1/2) [30].

The advantage of using receptor mutants is that disruption of endocytic trafficking is specific for the EGFR. However, often to test the mutant receptors, they are placed in cells that do not contain endogenous EGFRs and therefore have an unnatural signaling environment. Further, when expressing these receptors in cells, it can be difficult to achieve physiological (or pathologically) meaningful receptor densities.

### 4.2. Inhibitors of the Endocytic Pathway

A second approach for dissecting the role of the endocytic pathway is to inhibit the endocytic pathway directly. This can be done with pharmacologic and genetic reagents, or by reducing the cell’s temperature. One overarching concern with this type of approach is that other cargo that share this pathway have blocked endocytic trafficking as well. Therefore, there needs to be caution in interpreting whether the consequences are specifically due to changes in EGFR trafficking, another receptor, or a change in a cellular process.

Pharmacologic agents can be used in most cell lines and their effects are relatively rapid. However, there can be questions regarding the exact endocytic stage that is being blocked due to off target effects. Examples of drugs that can block endocytic trafficking include monensin, bafilomycin, chloroquine [42–44]. These drugs work by preventing acidification of endocytic compartments and impairing subsequent membrane trafficking.

The genetic approaches target proteins that have previously been demonstrated to regulate a specific endocytic stages and inhibiting their function by expressing a dominant negative form or ablating their expression with RNA interference (RNAi). In general, this strategy is very focused in terms of which step in the endocytic pathway is blocked. Prior to the wide spread use of RNAi, dominant negative proteins were the main mechanism for disrupting trafficking [19,45]. One of the advantages of dominant negative proteins is that they can inhibit the function of multiple homologs of the same gene. For instance, expression of dominant negative mutant dynamin I can inhibit the function of dynamin II despite tissue-specific differences in protein distribution [46]. Dominant negative form of canine rab5 can inhibit the activity of all three human RAB5 proteins (RAB5a, RAB5b, RAB5c), presumably due to the >97% homology between species [47].

The recent advances in RNAi technology have made knock down approaches more common. RNAi targets only a specific gene and the introduction of siRNAs and shRNAs are relatively easy. Although in some cases, all functional homologs need to be knocked down to achieve a complete effect, that typically is not an insurmountable obstacle. In fact, depending on the research goal, knocking down individual homologs may be more desirable [48].

While the genetic strategies offer more specific targeting of an endocytic stage, they introduce a temporal confounder to the experiment. Typically, it takes 48–72 h for either the dominant negative protein to be fully expressed or for maximal knock down of the target protein. Between the time of dominant negative protein cDNA or gene specific RNAi is introduced to the cell and the experiment, there is a continuum of inhibited endocytic trafficking. This will alter the dynamic endocytic pathway and change the steady-state composition, as well as allow compensatory mechanisms to arise.

An analogous strategy is the reduction of temperature. Maintaining the cell temperature at 4 °C will block endocytosis; keeping the cell at 22 °C will block intracellular trafficking [49]. This approach effectively reduces endocytic trafficking but the associated reduction in effector activity precludes a meaningful analysis of signaling.

### 4.3. Immobilized Ligands

The third strategy that has been used to study the relationship between EGFR signaling and trafficking has been the choice of ligand. While Mother Nature has provided ligands that differentially effect EGFR endocytic trafficking (highlighted by Roepstorff *et al.* [50]), they do not restrict the ligand:receptor complex to a single location.

A more precise way to disrupt trafficking entails using a ligand-bound matrix that is too large to internalize. This has been done using polystyrene beads [51–53] as well as tissue culture dishes [54]. This approach has the advantage that any EGFR expressing cell line can be studied. Further, there is little risk of any compensatory mechanisms occurring with immobilized ligands, as receptor responses can be measured as soon as the ligand is presented. It turns out this may be a biologically relevant mode of EGFR signaling. All of the naturally occurring EGFR ligands are made as “pro-growth factors” that must be cleaved from their tethered, membrane-bound form prior to becoming a diffusible ligand. Experiments, that will be discussed in detail later, have shown that EGFR activation by a tethered ligand is different than a soluble one [55]. Although it has yet to be demonstrated this occurs under physiological conditions, it is likely that nature has its own tethered ligand.

Synthetic immobilized ligands do have some caveats. First, the binding properties are likely not the same as unmodified EGF. Normally, EGF binds to each of the EGFR monomers in a sequential fashion. First one molecule binds a EGFR monomer with high affinity, that ligand:receptor complex binds another EGFR monomer, which finally binds a second EGF molecule with lower affinity [56]. It seems unlikely this sequence of events is recapitulated when receptors are stimulated with immobilized ligands and the movement of ligand is sterically hindered. However, recent crystallographic studies indicate that binding of only one ligand is sufficient to activate and an EGFR dimer [57]. Therefore, this may be an irrelevant concern.

Second, it can be difficult to truly assess the concentration of ligand to which the cells are being exposed. Much depends on the efficiency of ligand conjugation to the matrix. In addition, by the nature of the immobilization, the ligands will not diffuse with Brownian motion the way soluble ligands do. This means there are elevated localized levels of the ligand at the cell surface, which may or may not be favorable for signaling.

## 5. Recent Findings

To the uninitiated observer, the strategy for studying endocytic regulation of EGFR signaling seems straight forward: take cells with and without blocked endocytosis, treat with EGF, and look for differences in the activity of downstream effectors. However, the reality is much more complex. Two major concerns among the scientists that do these studies are: “what signaling events are being overlooked?” and “what signaling events are physiologically relevant?” The specifics of which cell line, EGFR density, EGF concentration, and endocytic inhibitor to use, and which effectors to examine all contribute to the problem. Each of these factors can affect the outcome and result in overlooking important biochemical and biological changes.

### 5.1. Proteomic Approach

Recently, Omerovic *et al.* published a report using an unbiased examination of phosphoproteins whose activity was affected by endocytosis of the EGFR. Briefly, they blocked EGFR endocytosis in HeLa cells with a pharmacologic inhibitor of dynamin, Dynasore, and assessed the generation of phosphospecific proteins generated in response to EGF [58]. This is a noteworthy experiment because (1) the method of phosphoprotein detection, Stable Isotope Labeling of Amino acids in Cell culture or SILAC, contains internal controls for each treatment and effector and (2) the approach is unbiased in its examination of effectors. In this study, the authors were able to compare the level of phosphorylation of hundreds of proteins and report that >40% of the EGF-dependent protein phosphorylation was effected by receptor endocytosis. Both regulators of EGFR endocytic trafficking and signaling proteins were well represented among the affected proteins.

The breadth of EGFR signaling that is spatiotemporally regulated is not surprising to those of us in the field and this study provides plenty of data to keep us excited. Despite being an unbiased approach, this study also brings us back to the questions regarding doses of EGF, time point, endocytic stages, *etc*. Testing these variables is not possible with this data intensive approach. Nevertheless, this study provides an opportunity for others that study the EGFR (and other receptor tyrosine kinases) to see if receptor:effector communication of their favorite phosphoproteins are affected by endocytosis. Two critical questions that remain are: (1) Which of these interactions are physiologically relevant? and (2) Is the spatialtemporal regulation of signaling cell-type specific, or is it independent of the concentrations of receptors, effectors, and scaffolding proteins?

### 5.2. Effector Focused Approach

The majority of early studies of the spatial regulation of EGFR signaling focused on receptor:effector communication. That is, examining the activity of one or two specific effectors using dose response curves and time courses under endocytosis-deficient and endocytosis-permissive conditions. This strategy has been used to examine effectors such as: STAT3 [59], p21(ras) [60], and phospholipase Cγ1 [61]. However, one of the most well studied has been the mitogen-activated protein kinase (MAPK)/extracellular signal-regulated kinase (ERK1/2) pathway. ERK1/2 is a serine/threonine kinase stimulated by the Ras-Raf-MEK-ERK1/2 signaling cascade that is conserved from yeast to mammals. It has an established role in cellular DNA and protein synthesis and, like the EGFR, has important developmental and homeostatic roles (Reviewed in [62]).

In addition to the established physiological roles, ERK1/2 is of interest because it was among the first proteins identified as having a spatiotemporal regulation to its signaling [19,63]. Although not all reports are in agreement, the majority of literature indicates that ERK activation by EGFR is spatially regulated. These data come from a variety of studies that block receptor internalization using mutant EGFRs that are defective in endocytic trafficking and the targeted inhibition of the endocytic pathway with dominant negative proteins or RNAi.

Despite the data indicating the full activation of ERK1/2 and MEK requires EGFR endocytosis, that may just be the start of the story. It should be noted there is evidence that not only cell surface receptors move about the cell during endocytosis, but the effector proteins do as well. Work by Galperin and Sorkin provide evidence that the effector, MEK, moves into endosomes when the EGFR undergoes endocytosis [64]. In their study, they demonstrate that the loss of clathrin-dependent endocytosis will decrease the recruitment of activated MEK into the endosome, despite no loss in total EGFR-stimulated MEK activity. When ERK activity was examined, blocking endocytosis resulted in enhanced ERK1/2 activity. The authors postulate that endosomal localization of MEK may be part of a negative feedback loop.

This begs the question: How does this occur? An idea that is growing in popularity is that scaffolding proteins may be critical in conferring this regulation. Scaffolding proteins for the ERK1/2 signaling cascade were first discovered in yeast [65] and are present in higher order organisms as well. By tethering multiple proteins together, they are a feasible means of moving a signaling cascade through the endocytic pathway together to provide spatial regulation of effector activity. The scaffolding protein Shoc2 [66], tethers the RAS-RAF-MEK signaling cascade [67]. Galperin *et al.* recently demonstrated that Shoc2 moves along the endocytic pathway to RAB7 positive late endosomes upon EGFR stimulation. Despite being an intracellular protein, knock down of clathrin to prevent endocytosis of the liganded EGFR, prevents Shoc2 intracellular movement. This is consistent with Shoc2 being trafficked with the receptor. Both the loss of Shoc2 (by RNAi) and certain mutants of Shoc2 that lead to membrane targeting result in decreased ERK1/2 activity [66]. These data provide strong evidence that Shoc2 is a spatial and temporal regulator of EGFR-mediated ERK1/2 signaling. However, further studies are needed to determine how this regulation affects the cell biology.

### 5.3. Transcription Factors

To understand the biological consequence of EGFR endocytic trafficking, several groups have looked even further downstream of the receptor and examined the activity of transcription factors [68,69]. Work by Wu *et al.* found that EGFR-mediated activation of transcription factors was spatially regulated [69]. They examined signaling by a mutant EGFR that was retained at the plasma membrane or by EGFRs that were pharmacologically manipulated to only signal from endosomes. The authors report that despite comparable levels of ERK1/2 activity produced by both receptor populations, the plasma membrane receptors preferentially induced phosphorylation of the transcription factor, c-fos, but not c-jun. Conversely, active EGFRs in endosomes specifically phosphorylated ELK. Together, these studies support the idea that there may be two (or maybe more) distinct populations of ERK1/2 in the cell that are activated from distinct endocytic locales.

In contrast to the focused examination of transcription factors, another group engaged in a more global and unbiased study. Brankatschk *et al.* treated endocytosis-deficient cells with and without EGF and examined mRNA levels by microarray [68]. In addition to concluding transcriptional activity was initiated soon after receptor stimulation, they identified 263 genes whose expression was differentially regulated by the endocytic process. While there are a number of more detailed studies that are needed to sort through the significance of all differentially regulated transcripts, this study provides an important starting point because endocytic trafficking was disrupted using multiple approaches in three different cell lines. Therefore, rigorous criteria for transcriptional regulation can be used.

### 5.4. Cell Biology Approach

In recent years, a greater emphasis has been placed on understanding the biological consequences to disrupting EGFR endocytosis. How does the spatial placement of the EGFR affect the cell’s function? Although the EGFR is associated with cell proliferation, survival, and migration, there is a well-established link between EGFR signaling and apoptosis. The subcellular location of the activated receptor may determine whether the EGFR signals cells to cell growth *versus* death.

The notion that EGFR-mediated apoptosis is spatial regulated was introduced by Singh *et al*., and their work on kidney epithelial cells. Briefly, they examined EGFR signaling in response to heparin binding-EGF (HB-EGF). HB-EGF, like all EGFR ligands, is synthesized as a membrane-tethered ligand that becomes cleaved into a soluble form that can diffuse and bind a receptor. To determine whether the membrane bound form of HB-EGF could still signal to the EGFR, they engineered cells to express a non-cleavable, membrane anchored form of HB-EGF [70]. They found that not only could the membrane bound ligand bind to and activate the receptor, but affected cell biology differently than the diffusible ligand. When the ligand:receptor complex was tethered to the plasma membrane it promoted cell proliferation; when internalized the result was apoptosis [55].

In a similar line of investigation, Hyatt and Ceresa compared the signaling by soluble EGF and immobilized EGF (EGF covalently linked to polystyrene beads) to assess the spatial regulation of EGFR signaling in MDA-MB-468 cells [71]. Like the study by Singh *et al.*, they found that when the activated receptor was retained at the cell surface receptor, cell growth was promoted and intracellular receptors induced apoptosis.

Work by Rush *et al.* provides further insight into how intracellular receptors can induce apoptosis. They postulated that the high levels of EGFRs on MDA-M-468 cells (~1.6 × 10^6^ EGFRs/cells [72]) contributed to both defects in intracellular, endocytic trafficking, and the induction of apoptosis. They had found that when MDA-MB-468 cells were treated with EGF, the EGF:EGFR complex accumulated on the limiting membranes of the endosomes. The orientation was such that the receptor’s phosphotyrosines were accessible to cytosolic downstream effectors. They went on to demonstrate that cells with low levels of EGFRs (HeLa cells) could be engineered to undergo EGF-dependent apoptosis if endocytic trafficking were blocked by knocking down the ESCRT protein TSG101 and the receptor orientation on the limiting membrane of the endosome was recapitulated. Importantly, when trafficking was blocked and the EGFR accumulated in the intraluminal vesicle of the late endosome/multivesicular body, there was no induction of apoptosis [73]. Thus, cells could be engineered to produce a high enough concentration of EGFRs in endosomes to induce apoptosis.

This finding is reminiscent of what has been reported by Overmeyer *et al.* [74]. In this study, they examined the oncogene Ras and find that its accumulation in vacuoles leads to a necrosis-like cell death called “methuosis”. Although there are some major experimental differences, it is difficult to ignore the similarities. Like the spatially regulated active EGFR, Overmeyer *et al.* show that accumulation of a signaling molecule on the membrane of an intracellular organelle which ultimately catastrophic for the cell. Despite the differences, the end result is the same. In both cases, a biological significance of these pathways remains unclear. Nevertheless, both findings may provide new insights into how to target cancer cells for destruction. More recent studies have identified molecules that can recapitulate methuosis, with the goal of using them as part of an anti-cancer therapy [75,76].

## 6. Conclusions

For over 25 years, the spatiotemporal regulation of EGFR signaling has been studied. The field has evolved from viewing endocytosis as strictly a negative regulator of the ligand:receptor complex to now appreciating its complexity in both positively and negatively modulating receptor:effector communication.

Early studies focused on identifying the proteins that coordinate the active EGFR through the endocytic pathway, with an eye on inhibiting the activity of those proteins or disrupting interaction and trafficking of the receptor. Although it is likely not every regulatory protein of EGFR endocytic trafficking has been identified, a sufficient number have to provide a set a tools that allow us to redirect our efforts to the next step: understanding how endocytic trafficking affects EGFR-dependent changes in cell biology.

While it seems intuitive to unravel the complexity of EGFR signaling at the level of receptor:effector interactions, it is not easy. The EGFR has been shown to interact with countless downstream effectors, many of which interact in a manner that is dependent on receptor density or cell context. Looking for changes in cell biology may be the next step, but this is not trivial. Manipulating the endocytic pathway is best done with tissue culture model, which often have only subtle biological responses to EGFR activation. Further, naturally occurring changes in EGFR trafficking often do not really manifest themselves (*i.e.*, cancer) until later in life. This many suggest either very minor biological changes that amplify overtime or that many cells develop compensatory mechanisms to accommodate such changes.
